# Association Between Depressive Symptoms and Comorbidities Among Elderly Diabetic Individuals in China

**DOI:** 10.1002/brb3.70232

**Published:** 2024-12-31

**Authors:** Luyao Qiao, Xin Pan, Tianpei Li, Shouqin Yi, Zhenyu Tang

**Affiliations:** ^1^ Department of Neurology, the Second Affiliated Hospital, Jiangxi Medical College Nanchang University Nanchang Jiangxi China; ^2^ The Second Department of Neurology Jiangxi Provincial People's Hospital, Clinical College of Nanchang Medical College, First Affiliated Hospital of Nanchang Medical College Nanchang Jiangxi China

**Keywords:** CHARLS, comorbidity, depressive symptoms, diabetes mellitus

## Abstract

**Background:**

Diabetic individuals are at an increased risk of mental illness and comorbidities. However, the precise association between depressive symptoms and comorbidity remains uncertain. Our study aimed to investigate this relationship among elderly Chinese diabetic patients.

**Methods:**

Data from the China Health and Retirement Longitudinal Study (CHARLS) in 2020 were utilized for the cross‐sectional analysis. Depressive status was defined as the dependent variable, while the presence, number, and type of comorbidities served as independent variables. Logistic regression analyses were performed, adjusting for potential demographic factors, and health status and functioning factors.

**Results:**

Our findings indicate that diabetic patients with complications are more likely to experience depression. With the exception for dyslipidemia (OR = 1.195, 95% CI: 0.969, 1.475), individuals with hypertension, heart disease, stroke, kidney disease, memory‐related disease, or arthritis/rheumatism were prone to develop depressive status in the fully adjusted model. After adjusting for covariates, diabetic patients with memory‐related diseases exhibited the most pronounced association with depressive symptoms (OR = 2.673, 95% CI: 1.882, 3.797). Furthermore, an increasing number of depression‐related comorbidities strengthened the association (*p* < 0.05). Sensitivity analysis revealed that there were no significant differences stratified by gender or marital status (*p* < 0.05).

**Conclusions:**

In the elderly diabetic population in China, the presence, number, and type of comorbidities were independently associated with depressive symptoms. Diabetic patients with memory‐related diseases displayed the highest likelihood of experiencing depressive status. These findings underscore the importance of implementing effective strategies for multimorbidity management in diabetic patients.

## Graphical abstract

The relationships between various diabetes‐related complications and depressive status. Memory‐related diseases have the most significant impact on depressive symptoms, while dyslipidemia shows no association among these complications.

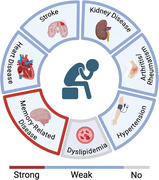

**Abbreviations**
CESD: Center for Epidemiological Studies Depression
CHARLS: China Health and Retirement Longitudinal Study
CI: confidence interval
DM: diabetes mellitus
OR: odds ratio
T1DM: Type 1 diabetes mellitus
T2DM: Type 2 diabetes mellitus

## 1 Introduction

The International Diabetes Federation (IDF) Virtual Congress 2023, held from December 4 to 7, provided the latest updates on diabetes management and its associated complications. The Congress highlighted that globally 540 million individuals are affected by diabetes (International Diabetes Federation 2024), with 1 in 10 people living with the condition (International Diabetes Federation 2023). Diabetes is emerging as the most prevalent epidemic disease, with projections indicating a staggering rise to approximately 783 million diabetic patients by 2045 (International Diabetes Federation 2024; Ogurtsova et al. 2022). However, nearly half of these individuals are unaware that they have this condition. Prolonged hyperglycemia and metabolic disorders can result in chronic tissue and organ damage, notably impacting the cardiovascular, immune, and central nervous systems, thereby imposing substantial financial burdens on both individuals and society (American Diabetes Association Professional Practice Committee 2023).

With the growing awareness of mental health status, depression among elderly people has been identified as a serious public health concern. Physical discomfort often overshadows emotional symptoms in older people. Moreover, compared to the general population, individuals with diabetes mellitus (DM) have a much higher risk of depression (Moulton, Pickup, and Ismail 2015). Tapash Roy's systematic review underscores that the incidence of depression is more than threefold greater in individuals with Type 1 diabetes mellitus (T1DM) and almost twice as high in those with Type 2 diabetes mellitus (T2DM), than in those without (Roy and Lloyd 2012). A large representative community‐based study of an elderly population in Spain confirmed that diabetes is linked to an elevated risk of depression among middle‐aged and older adults, regardless of diabetes type (de Jonge et al. 2006).

Furthermore, individuals with diabetes are prone to comorbidities, exacerbating the burden on depressed diabetics through impaired metabolic regulation, heightened vascular complications, disability, decreased quality of life, higher healthcare expenditures, and elevated mortality risks (Heckbert et al. 2010; Lin et al. 2010). It is also known that complications can precipitate depressive symptoms (Hargittay et al. 2023), further complicating the elucidation of correlations. However, the precise mechanisms underlying the association between diabetes and depressive symptoms remain elusive, with conflicting conclusions regarding the role of comorbidity. It had been reported by Antonio Campayo that depression incidence seemed to be elevated among those with diabetes comorbidity (Campayo, Gómez‐Biel, and Lobo 2011). Conversely, a Zaragoza Dementia and Depression (ZARADEMP) project studied the cross‐sectional and prospective associations of DM and major depression, and suggested that disability and comorbidity appear to mitigate the effect of diabetes on the likelihood of prevalent depression (de Jonge et al. 2006).

Consequently, in addition to common diabetic comorbidities, we incorporated factors that may influence the occurrence of both diabetes and depressive symptoms, including demographic characteristics and health status, consistent with previous research findings (Collins, Corcoran, and Perry 2009; Roy and Lloyd 2012). This investigation utilized data from the China Health and Retirement Longitudinal Study (CHARLS) data to investigate: (1) the correlation between depressive symptoms and the presence and number of comorbidities in individuals with diabetes, (2) which diabetic complications are associated with the prevalence of depressive status, and (3) whether the link between depressive status and the presence of any comorbidity varies in sensitivity analyses.

## 2 Methods

### 2.1 Study Population

The CHARLS, a nationally representative longitudinal survey of people living in 28 provinces, municipalities, and autonomous areas who are 45 years of age or older along with their spouses, served as the basis for this investigation. An abundance of information is available through CHARLS, such as demographics, health and functioning, employment and pension, and more (Zhao et al. 2014). We accessed the CHARLS database online shortly after the release of the 5th wave (2020) and obtained approval quickly. Part of the data, such as education and medical history information, were from the previously available waves.

**FIGURE 1:**
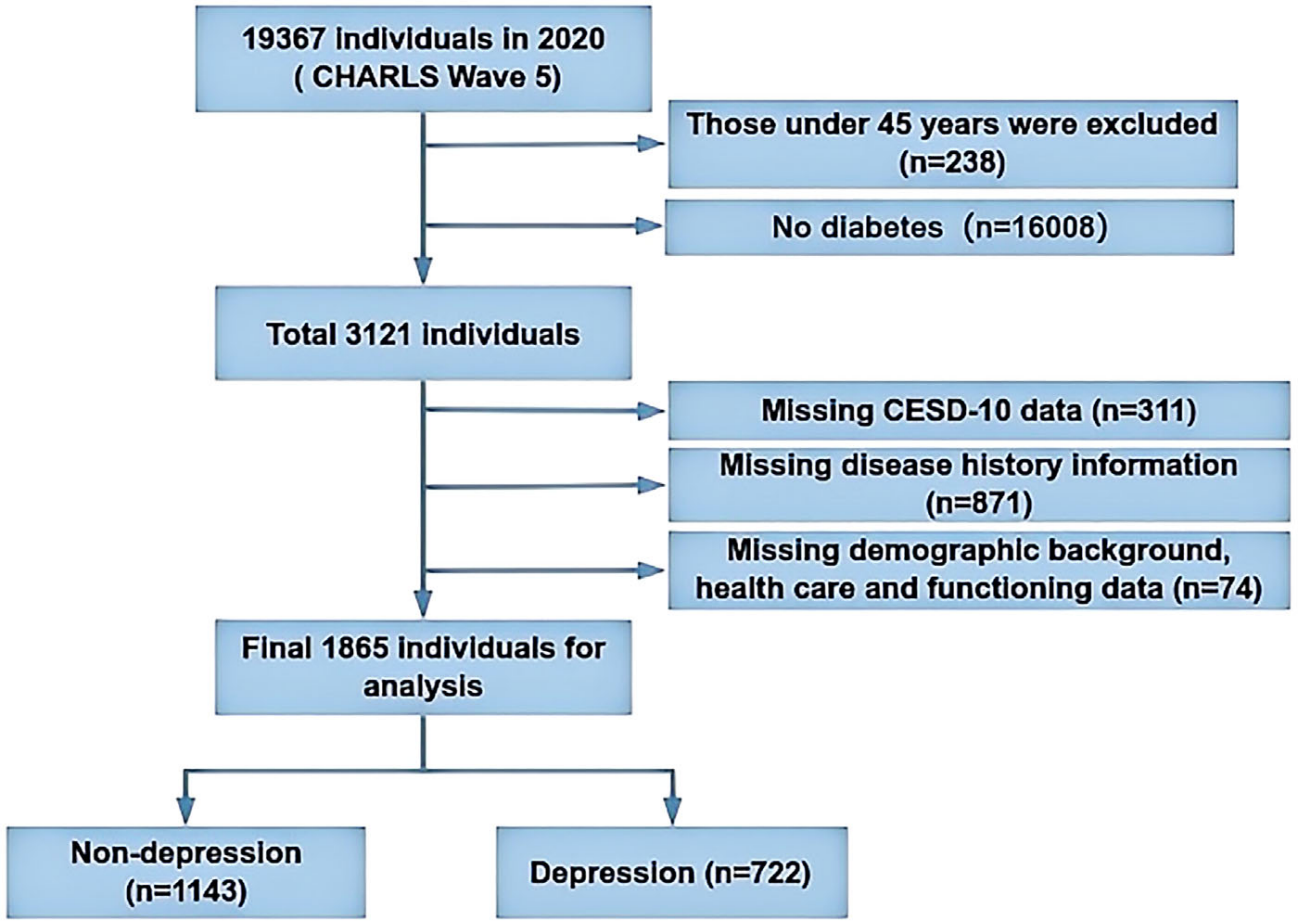
Flowchart of this study's individuals selection.

Based on the purpose of our study, the subjects fulfilled the criteria of being over 45 years old and having been diagnosed with DM. Participants who were missing the 10‐item Center for Epidemiological Studies Depression (CESD‐10) scores and had incomplete demographics, health status, and cognition information were excluded. Figure 1 depicts a thorough flowchart of the participant selection procedure. Eventually, this cross‐sectional analysis included 1865 participants.

### 2.2 Defining Depressive Symptoms

The CESD‐10, used to screen depressive symptoms in our study, is derived from the full‐length 20‐item CESD and has better predictive accuracy (Andresen et al. 1994). The participants were asked to rate their frequency of encountering every item during the course of the preceding week. The items ranged from 0 (rarely or never) to 1 (some of the time), 2 (much or a moderated amount of the time) to 3 (most or all of the time) for the most part. However, two of the scale's positive items were inverted. Higher scores correspond to more severe symptoms of depression. Previous research has proven that a cut‐off point of 10 has shown good sensitivity and specificity in elderly Chinese (Andresen et al. 1994; Cheng and Chan 2005). In our study, those who scored a CESD‐10 of 10 or more were classified as having depressive status.

### 2.3 Defining Diabetes‐Associated Comorbidities

Based on the CHARLS questionnaire and related studies (ElSayed et al. 2022; Markle‐Reid et al. 2018), diabetes‐associated comorbidities included hypertension, dyslipidemia (high or low blood lipids), heart disease (e.g., myocardial infarction, coronary heart disease, angina pectoris, congestive heart failure, and other heart diseases), stroke, kidney disease (except for tumor or cancer), memory‐related disease, and arthritis/rheumatism. Every participant in CHARLS was asked, “Have you been diagnosed with the conditions listed above by a doctor?” If someone answered “yes,” they were assumed to have the disease and recorded as 1; if not, then as 0. The participants' number of comorbidity was the sum of the above complication scores. If the total score was 0, he had no comorbidities.

### 2.4 Covariates

According to prior knowledge, we also considered demographic background, health status, and functioning in our study. Demographic background factors included age, gender, residential area (urban or rural), education (illiterate, primary school and below, or junior high school and above), marital status (married/partnered or unmarried/separated/divorced/widowed), and social endowment insurance (pension insurance for employees of state organs or public institutions, worker's basic endowment insurance, social endowment insurance for nonworking urban residents, new social endowment insurance for rural residents, social endowment insurance for urban and rural residents, or other). The indicators of health status and functioning were the following: regular exercise (high or moderate intensity activity lasting more than 30 min per day and more than 3 days per week, or not) (Dong et al. 2023), smoking and drinking status (still performing or never/quit), and cognitive scores.

### 2.5 Measurement of Cognitive Function

The assessment of cognitive ability involved measuring episodic memory, orientation, attention, and visuospatial skills (Rawtaer et al. 2017). Ten questions from the telephone interview of cognitive status were used to primarily measure orientation and attention (Fong and Inouye 2018), including date (month, day, year, and season), day of the week, subtracting 7 from 100 (up to five times), with 1 point for each correct question. Word recall tests were used to assess episodic memory (Lei et al. 2014). The score was the average of the correct immediate (within 2 min) and delayed (after 4 min) recall words. Respondents were asked to draw the displayed figure to assess visuoconstruction (Huang and Zhou 2013; Mathuranath et al. 2000). All of these dimensions, when added together, yielded an overall cognitive score that ranged from 0 to 21. Higher scores indicate better functioning.

### 2.6 Statistical Analysis

SPSS 26.0 and STATA V17.0 were used to accomplish all statistical analyses. Categorical variables were formatted as a number (percentage). The continuous variables were presented as medians (interquartile ranges) due to their non‐normal distribution. The Mann–Whitney *U* test or the chi‐squared test was used to compare the characteristics of the individuals. We initially conducted univariate regression analyses to assess the independent association of each comorbidity with depressive symptoms. Subsequently, we used three multivariate logistic regression models that were adjusted for various confounding factors to look at the connection between depression and comorbidity among these participants. Finally, we performed sensitivity analyses stratified by gender and marital status to evaluate the reliability of the results. The level of significance was < 0.05.

## 3 Results

From the total of 1865 participants with diabetes aged ≥ 45 years who were included in this study, there were 722 participants with depressive symptoms and 1143 without depressive symptoms. Except for regular exercise (*p* = 0.660), all variables were significantly associated with depressive status (*p* < 0.05). Participants who are women, aged 60–75 years, with primary school and below education, not currently smokers or drinkers, living in a rural area, living alone, and having rural health insurance were more likely to get depressive symptoms (Table 1).

**TABLE 1:** Baseline characteristics of participants by depressive status.

Variates	Total	Nondepression	Depression	*p* value
**Gender**				
Female	971 (52.1)	515 (45.1)	456 (63.2)	< 0.001
Male	894 (47.9)	628 (54.9)	266 (36.8)	
**Age (year)**				
< 60	738 (39.6)	474 (41.5)	264 (36.6)	0.023
60–75	946 (50.7)	551 (48.2)	395 (54.7)	
≥ 75	181 (9.7)	118 (10.3)	63 (8.7)	
**Education**				
Illiterate	351 (18.8)	165 (14.4)	186 (25.8)	< 0.001
Primary school and below	769 (41.2)	449 (39.3)	320 (44.3)	
Secondary school or higher	745 (39.9)	529 (46.3)	216 (29.9)	
**Smoking status**				
Current smoker	417 (22.4)	297 (26.0)	120 (16.6)	< 0.001
Nonsmoker	1448 (77.6)	846 (74.0)	602 (83.4)	
**Drinking status**				
Current drinker	628 (33.7)	430 (37.6)	198 (27.4)	< 0.001
Nondrinker	1237 (66.3)	713 (62.4)	524 (72.6)	
**Residence**				
Urban	838 (44.9)	557 (48.7)	281 (38.9)	< 0.001
Rural	1027 (55.1)	586 (51.3)	441 (61.1)	
**Marital status**				
Married/partnered	1596 (85.6)	1005 (87.9)	591 (81.9)	< 0.001
Unmarried/separated/divorced/widowed	269 (14.4)	138 (12.1)	131 (18.1)	
**Regular exercise**				
Yes	854 (45.8)	528 (46.2)	326 (45.2)	0.660
No	1011 (54.2)	615 (53.8)	396 (54.8)	
**Social endowment insurance**				
Pension insurance for employees of state organs or Public institutions	398 (21.3)	303 (26.5)	95 (13.2)	< 0.001
Worker's basic endowment insurace	172 (9.2)	108 (9.4)	64 (8.9)	
Social endowment insurance for non‐working urban residents	108 (5.8)	68 (5.9)	40 (5.5)	
New social endowment insurance for rural residents	1127 (60.4)	621 (54.3)	506 (70.1)	
Social endowment insurance for urban and rural residents	30 (1.6)	26 (2.3)	4 (0.6)	
Other	30 (1.6)	17 (1.5)	13 (1.8)	
**Cognitive scores**	11.5 (8.0, 14.5)	12.0 (9.0, 15.0)	9.5 (6.5, 12.5)	< 0.001^*^
**CESD‐10**	7.0 (4.0, 13.0)	4.0 (2.0, 7.0)	15.0 (12.0, 19.0)	< 0.001^*^

* Mann–Whitney *U* test, continuous data are represented as M (P25, P75).

Abbreviation: CESD, Center for Epidemiologic Studies Depression.

Table 2 shows the differences in the presence, number, and type of comorbidities and depressive status classified by status of depression. The presence and the number of comorbidities differed significantly between diabetics with and without depressive status. Hypertension, dyslipidemia, heart disease, stroke, kidney disease, memory‐related disease, and arthritis/rheumatism were significantly more common in diabetic individuals with depression than in those without (*p* < 0.05).

**TABLE 2:** Comparison of the presence, number, and type of comorbidities between depression and non‐depression, *N* (%).

		Nondepression	Depression	χ^2^	*p* value
**Any comorbidities**	Yes	945 (82.7)	658 (91.1)	26.219	< 0.001
	No	198 (17.3)	64 (8.9)		
**Number of comorbidities**	0	198 (17.3)	64 (8.9)	122.923	< 0.001
	1	362 (31.7)	156 (21.6)		
	2	314 (27.5)	182 (25.2)		
	3	175 (15.3)	155 (21.5)		
	≥ 4	94 (8.2)	165 (22.9)		
**Type of comorbidities**	Heart disease	352 (30.8)	294 (40.7)	19.249	< 0.001
	Stroke	99 (8.7)	123 (17.0)	29.594	< 0.001
	Kidney disease	140 (12.2)	185 (25.6)	55.009	< 0.001
	Hypertension	689 (60.3)	494 (68.4)	12.643	< 0.001
	Dyslipidemia	336 (29.4)	253 (35.0)	6.526	0.011
	Memory‐related disease	63 (5.5)	104 (14.4)	42.921	< 0.001
	Arthritis or rheumatism	256 (22.4)	289 (40.0)	66.500	< 0.001

**FIGURE 2:**
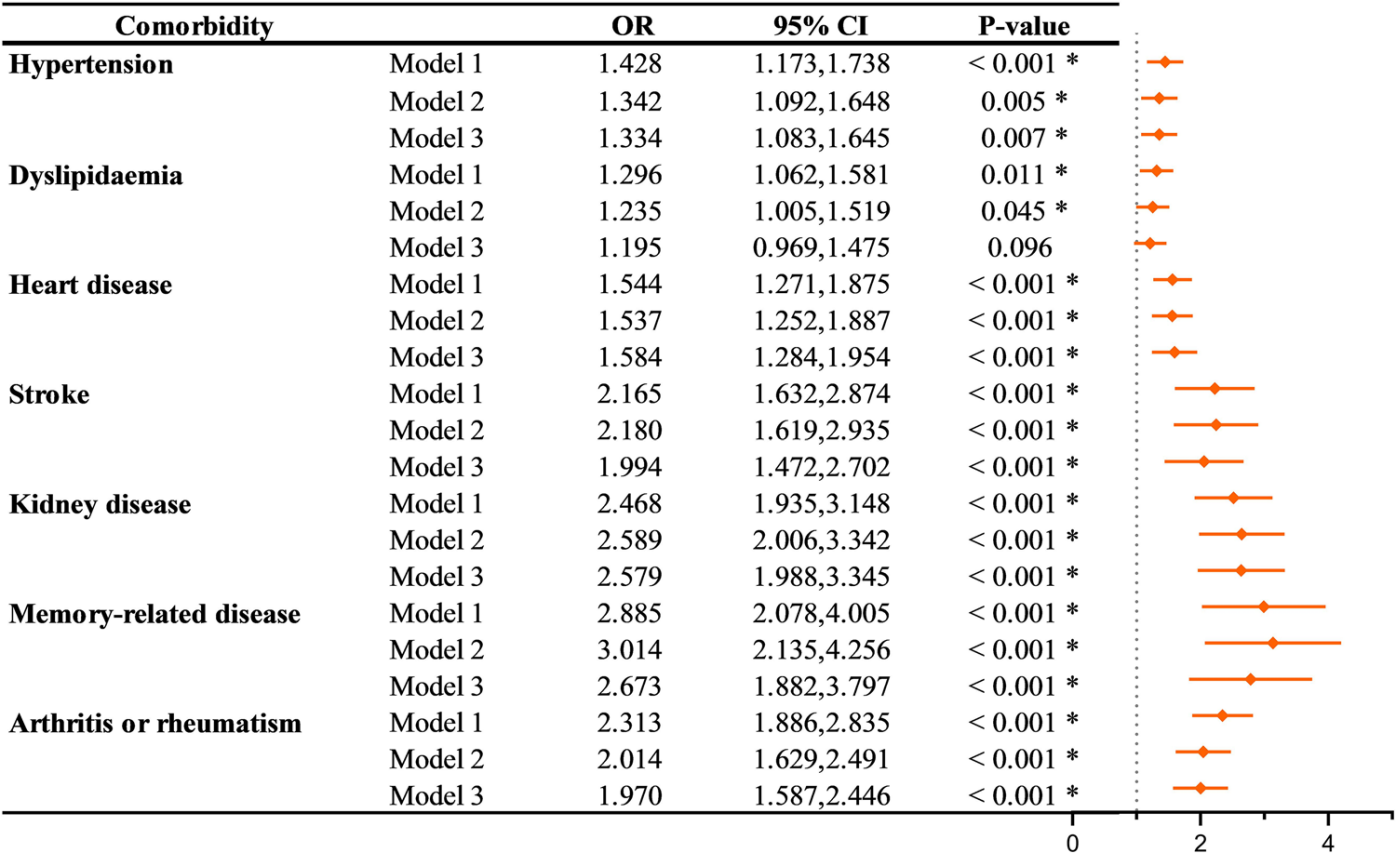
Association between depression with each diabetic comorbidity. Model 1: no adjustment; Model 2: adjusted for demographic background factors (including age, gender, residential area, education, marital status, and health insurance); Model 3: adjusted as per Model 2 and with health status and functioning factors (including smoking and drinking status, regular exercise, and cognitive scores). *p < 0.05. 95% CI: 95% confidence interval; OR: odds ratio.

**FIGURE 3:**
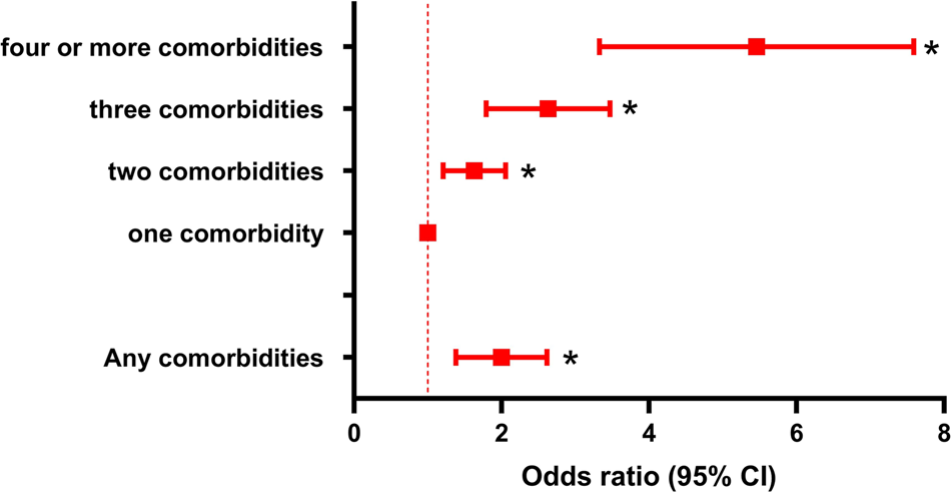
Association between the status, number of comorbidities related to depression and depression. ORs were adjusted for age, gender, residential area, education, marital status, health insurance, smoking and drinking status, regular exercise, and cognitive scores. *p < 0.05. 95% CI: 95% confidence interval; OR: odds ratio.

As shown in Figure 2, we found that all comorbidities were associated with the incidence of depressive status in models 1 and 2. In the fully adjusted model, hypertension (OR = 0.334, 95% CI: 1.083, 1.645), heart disease (OR = 1.584, 95% CI: 1.284, 1.954), stroke (OR = 1.994, 95% CI: 1.472, 2.702), kidney disease (OR = 2.579, 95% CI: 2.006, 3.342), memory‐related disease (OR = 2.673, 95% CI: 1.882, 3.797), or arthritis/rheumatism (OR = 1.970, 95% CI: 1.587, 2.446) were respectively observed statistically significant with depressive status, with memory‐related diseases were the strongest associated with depressive status. Figure 3 shows that after fully adjusting for covariates, individuals with at least one diabetic comorbidity have a significant higher incidence of depressive status (OR = 1.933, 95% CI: 1.413, 2.645). Meanwhile, the increasing number of depression‐related comorbidities strengthened the correlation (two: OR = 1.593; 95% CI: 1.227, 2.069; three: OR = 2.541, 95% CI: 1.840, 3.509; and four or more: OR = 5.179, 95% CI: 3.477, 7.714). According to the sensitivity analysis grouped by gender and marital status, the correlations between the presence of comorbidities and depressive status remained unchanged (*p* < 0.05).

## 4 Discussion

We analyzed the relationship between depressive symptoms and the status, number, and type of comorbidity among the elderly diabetic population in this cross‐sectional investigation using the CHARLS wave 5 (2020) data. The results showed that people with DM, who had any complications, were more susceptible to depressive symptoms than those who did not have complications. This association was not influenced by gender and marital status. After adjustment for all the confounding factors, the development of depressive status was independently correlated with the following comorbidities: hypertension, heart disease, stroke, kidney disease, cognitive disorders, and arthritis/rheumatism, of which cognitive disorders had the strongest impact (OR = 2.673, 95% CI: 1.882–3.797). In addition, as the number of comorbidities increased, so did the risk of depression.

In multiple‐factor logistic regression analyses, including demographic background, health status, and functioning factors, we found that the relationship between depressive symptoms and middle‐aged and older diabetics can also be influenced independently by gender, age, marital status, social endowment insurance, and cognitive scores. Depressive symptoms were more common in people with diabetes who were women, the elderly, married or with a spouse, and had only rural medical insurance. This was similar to the previous studies. The majority of studies have found that age is a risk factor for depression (Golden et al. 2008; Mikaliūkštienė et al. 2014). A systematic study revealed that women with T1DM and T2DM had increased rates of depression than men (Roy and Lloyd 2012). The findings of our study confirmed that, when compared to single people, getting married or living with a partner made it much easier to acquire depression. This may be due to reduced long‐term family conflicts and heightened distress. Single people may have better emotional well‐being and pay more attention to themselves (Reese et al. 2010; Zhou et al. 2023). This highlights that it is a healthy and satisfying relationship that matters, not living with a spouse or partner. The type of social endowment insurance for individuals is linked to their employment, which partly reflects their economic status. For elderly individuals with rural social endowment insurance, their access to daily medical treatment may be limited (Zhou et al. 2023). Only when they have a serious illness do they seek treatment in a higher level hospital. Therefore, the daily management of blood glucose is easily neglected, which may lead to systemic complications.

The cognitive performance among older adults diagnosed with DM exhibited a noteworthy correlation with the prevalence of depressive symptoms. In the comprehensive adjusted model, the logistic regression analysis demonstrated that memory‐related disorder emerged as the most strongly associated comorbidity contributing to the development of depression. Memory‐related disorder is characterized by impairments in cognitive function, which includes conditions such as dementia, brain atrophy, and Parkinson's disease. These results agreed with prior studies, both cross‐sectional and longitudinal. A longtime longitudinal investigation of participants in the ACCORD‐MIND trial identified a strong connection between more severe mood disorders in T2DM patients and greater cognitive decline in all cognitive domain tests (Sullivan et al. 2013). Bordier et al. (2014) found that diabetic patients with cognitive deficits can compromise daily activities, thereby amplifying the prevalence of depression‐related symptoms. There was another study on the risk of depression and cognitive decline among individuals with T2DM (Ravona‐Springer et al. 2021). Compared to those without T2DM, an increase in depression symptoms over time is connected with simultaneous cognitive decline in older adults with T2DM. Cerebral small vessel disease may be the common underlying mechanism (Gorska‐Ciebiada et al. 2014). Meanwhile, decreased cognitive performance in DM patients has been identified as a precursor to poor diabetes control and inadequate emotion regulation, which fosters a cyclic relationship between depression and hyperglycemia (Black et al. 2018). Recently, a new concept called the “triad of impairment (TOI)” has been introduced, which consists of physical, cognitive, and emotional aspects. These diseases commonly coexist and demonstrate a reciprocal destructive relationship (Abdelhafiz, Davies, and Sinclair 2020).

Besides cognitive disorders, our study shows that hypertension, heart disease, stroke, kidney disease, memory‐related disease, and arthritis/rheumatism were all associated with an increased risk of depression, after controlling for all available confounders. As described in the literature, there are repeatedly comorbid relationships between depression, heart disease, and T2DM. T2DM has been found to be linked to an increased risk of cardiovascular disease (CVD) (Petrie, Guzik, and Touyz 2018). The co‐occurrence of these conditions in the same individual will lead to poorer outcomes, such as higher rates of adverse cardiovascular outcome events and worsened functional status. Conversely, psychological distress can also hurt heart disease outcomes (Rashid et al. 2023). Previous studies (Lustman, Griffith, and Clouse 1988; Mikaliūkštienė et al. 2014) showed that nephropathy has a direct impact on the depressive state of diabetic patients. One possible explanation can be that those who require dialysis or kidney transplantation have severe renal impairment. Regular follow‐up and significant lifestyle changes make them prone to greater mental stress (Wang et al. 2019). The data from the study of Mikaliūkštienė et al. (2014) confirmed that arterial hypertension (95% CI: 28.4–35.2), as a chronic complication of DM, affected the emotional state of patients and increased its prevalence by 31.7%. Another study conducted in Beijing found that depressive status was significantly more common in T2DM patients who had hypertension than in patients who had T2DM or hypertension alone (Deng, Li, and Zhang 2022). The results of a large, population‐based administrative cohort by Brown and his colleagues were consistent with us. Arthritis (HR = 1.18) and stroke (HR = 1.73) were linked to the prevalence of depression in patients with diabetes (Brown et al. 2006). Diabetes is characterized by chronic hyperglycemia and insulin resistance, which play a major role in the onset of microvascular and macrovascular diseases, including neuropathy, nephropathy, hypertension, and heart disease. These manifest vascular diseases’ effects of oxidative stress, inflammation, and cerebral perfusion abnormalities that can threaten the integrity of cerebral connections implicated in mood regulation (Blöchl et al. 2022; Willame et al. 2022). Arthritis/rheumatism is one chronic inflammatory disorder that could invade multiple tissues, organs, and systems, and cause physical disability. Meanwhile, neurocircuitry and inflammation are also intimately linked to the greater occurrence of depressive symptoms in these individuals (Minamino et al. 2021).

The mechanisms underlying the higher risk of depressive status in diabetic people are still unknown. It was often assumed that patients suffering from depression had an understandable and unresponsive reaction to any stimuli, which increased the burden of managing the condition and its problems connected with diabetes. Furthermore, it may be associated with hypothalamic‐pituitary adrenal (HPA) axis dysfunction (Champaneri et al. 2010; Ron Mizrachi et al. 2023), sympathetic nervous system (SNS) activation (Champaneri et al. 2010), chronic inflammation (Dantzer et al. 2008), insulin resistance (Kan et al. 2013), obesity, and environmental factors. Diabetics with any comorbidities are more strongly related to depressive affect than those without comorbidity. The more depression‐related comorbidities a person has, the greater the probability of depression is. This is in line with prior studies. A study published in *Diabetes Care* (Peyrot and Rubin 1997) highlighted the risk of depression and anxiety symptoms in adults with diabetes and resulted that diabetes increased the risk of psychological disease, especially in those with more diabetes‐related complications. This finding is also confirmed by a population‐based study in Germany (Icks et al. 2008), which discovered that comorbidities are highly related to depressed symptoms in both men and women with diabetes. Another longitudinal study (Fisher et al. 2008) demonstrated that having more comorbidities, as well as being younger and female gender, were related to affective and anxiety disorders, depressive affect, and diabetes distress in adults with Type 2 diabetes. Besides, they also found that there was a favorable relationship between the number of diabetic complications and depressive symptoms, which was similar to our result. Once having diabetes, it has a lifelong effect on the patients. They have to maintain stable blood sugar through a diabetic diet, exercising, and taking hypoglycemic drugs. But it is not easy. As the “3B Study” reported, the proportion of T2DM patients who met their target for blood glucose management (HbA1c < 7%) accounted for fewer than half (Ji et al. 2013). Through mechanisms such as insulin resistance, hyperglycemia, neuroinflammation, oxidative stress, and neuronal autophagy, diabetes is responsible for a heightened incidence of macrovascular and microvascular complications (Guo et al. 2016). Therefore, the negative effects of these complications on physical health and economic burden are doubled or accumulated in all patients with diabetes, which is related to the high rate of depression.

Nevertheless, the study has several limitations. First, our data were from the CHARLS database freshly released fifth wave. It was a cross‐sectional study. Failure to draw a causal relation may decrease the accuracy of results. Second, it is unclear whether people suffering from severe depressive symptoms can participate in completing the CESD‐10 scale, which introduces information bias. Finally, while we demonstrated a link between depressive status and the number of complications, we did not further analyze which comorbidities coexisting in patients with diabetes had the greatest impact on depression.

## 5 Conclusion

This cross‐sectional study shows well‐established evidence of a link between depressive symptoms and comorbidities in the elderly from China who have diabetes. Several diseases could increase the prevalence risk of depressive status in diabetic patients. The more comorbidities that were associated with depressive symptoms, the greater the chance of developing depression. It indicates that diabetes management should not only focus on glycemic control but also patients' mental health and comorbidity. These findings highlight the need for healthcare systems to transition from a single‐disease strategy to a more effective multimorbidity management model. This may help individuals with diabetic mellitus to prevent depressive status, manage with the load of multimorbidity, and improve their quality of life in middle and late adulthood.

## Author Contributions

**Luyao Qiao**: Writing—original draft; data curation; conceptualization; methodology. **Xin Pan**: Conceptualization; data curation; methodology; writing—original draft. **Tianpei Li**: Formal analysis. **Shouqin Yi**: Formal analysis. **Zhenyu Tang**: Writing—review and editing; funding acquisition.

## Ethics Statement

This study was granted by Peking University's Ethics Committee. The Institutional Review Board (IRB) approval number for the main household survey, including anthropometrics, is IRB00001052‐11015; the IRB approval number for biomarker collection is IRB00001052‐11014.

## Consent

Prior to participation, all subjects gave written informed consent.

## Conflicts of Interest

The authors declare no conflict of interests.

### Peer Review

The peer review history for this article is available at https://publons.com/publon/10.1002/brb3.70232.

## Data Availability

Our data that support the findings of this study are available from https://charls.charlsdata.com.
